# Call to action: cardiologists should promote influenza vaccination

**DOI:** 10.1007/s12471-021-01637-9

**Published:** 2021-10-14

**Authors:** G. L. Habib, H. Yousuf, J. Narula, L. Hofstra

**Affiliations:** 1 Department of Cardiology, Amsterdam University Medical Centres, location VU University medical center, Amsterdam, The Netherlands; 2 Icahn School of Medicine, Mount Sinai, New York, NY USA

**Keywords:** Influenza, Human, COVID-19, Global Burden of Disease, Vaccines, Cardiovascular Diseases

## Abstract

The COVID-19 pandemic has spurred clinical and scientific interest in the cardiology community because of the significantly enhanced vulnerability of patients with underlying cardiac diseases. COVID-19 vaccination is therefore of vital importance to the patients we see in our clinics and hospitals every day and should be promoted by the medical community, especially cardiologists. In view of vaccine-preventable diseases, the association between influenza and cardiovascular complications has been widely investigated. Several studies have found a substantially elevated risk of hospital admission for acute myocardial infarction in the first 7 days after laboratory-confirmed influenza, with incidence ratios ranging from 6.05–8.89. The effectiveness of the influenza vaccine to protect against acute myocardial infarction is about 29%. This effectiveness is comparable to or even better than that of existing secondary preventive therapies, such as statins (prevention rate approximately 36%), antihypertensives (prevention rate approximately 15–18%), and smoking cessation (prevention rate approximately 26%). As the influenza season is rapidly approaching, this Point of View article serves as a call to action: Cardiologists should promote influenza vaccination and actively advice their patients to get the seasonal influenza vaccination.

## COVID-19: looking through a novel lens

The COVID-19 pandemic has spurred clinical and scientific interest in the cardiology community because of the significantly enhanced vulnerability of patients with underlying cardiac diseases. Early studies have shown a five times higher mortality rate in patients with underlying heart disease compared with patients without heart disease [1]. One proposed mechanism of this increased vulnerability is the fact that the severe acute respiratory syndrome coronavirus 2 (SARS-CoV-2) enters the cells by binding to the angiotensin-converting enzyme 2 receptor, which is abundantly present in heart and blood vessels [2, 3]. COVID-19 vaccination is therefore of vital importance to the patients we see every day in our clinics and hospitals and should be promoted by the medical community, especially cardiologists.

## Influenza

In general, influenza viruses can cause acute respiratory illnesses, resulting in annual epidemics (seasonal) or less often pandemics. Annual epidemics are caused by influenza A and B viruses, whereas pandemics are caused by influenza A virus. The viruses can be transmitted from human to human in three ways: via aerosols, droplets and contact. When someone sneezes, coughs or talks, particles (aerosols and droplets) arise, which can then be inhaled by others. Contact transmission occurs via fomites (deposition of expelled virus particles onto surfaces) [4, 5].

The incubation period of influenza ranges between 1 and 4 days, and viral shedding peaks 2–3 days after the onset of sickness. Viral load and duration of viral shedding are higher in children than in adults [4]. In temperate regions, transmission occurs mostly seasonal (during the winter season), while it can take place all year round in tropical regions [5, 6]. Once infected, the manifestation of influenza may vary from mild sickness not requiring health services to severe illness, hospitalisation or even death. While the majority recovers within one week without medical care, elderly, infants, pregnant women, overweight individuals and patients with chronic underlying disease are more susceptible to a severe course of disease [6].

Today, seasonal influenza still leads to high morbidity and mortality worldwide [6–9], despite great advances in medical science and the development of vaccines [10, 11]. Specifically, low-income and middle-income countries are often disproportionately impacted by the virus [7], which in part could be fed by the low vaccine availability [12]. The Global Burden of Disease Study 2017 Influenza Collaborators calculated that 17.4% (9,459,000) of all patients with influenza-caused lower respiratory tract infections among all ages were hospitalised [7]. Lafond et al. showed that 14% of acute respiratory hospitalisations among adults globally are associated with influenza, which corresponds with an estimated 5 million hospitalisations per year [9].

### Influenza and the heart

Besides its involvement in respiratory tract infections, the burden of influenza carries broader consequences, including the effects on cardiovascular diseases (myocarditis, ischaemic heart disease, stroke) [13], chronic respiratory conditions (e.g. asthma exacerbation), diabetes mellitus (e.g. aggravating diabetic ketoacidosis or influenza complications due to chronic hyperglycaemia), neurologic complications (e.g. febrile seizures, influenza-associated encephalitis or encephalopathy, Guillain-Barré syndrome, exacerbations in patients with epilepsy), co-infections and secondary infections [6].

When focusing on cardiovascular complications, influenza epidemics have been associated with cardiovascular mortality for a long time [14]. A study from 2007 confirmed this relationship through autopsy-confirmed coronary deaths by showing a simultaneous peak in the number of patients with acute respiratory disease and influenza epidemics. During average influenza epidemic weeks, the odds for acute myocardial infarction (MI) and ischaemic heart disease amounted to 1.30 (95% confidence interval (CI) 1.08–1.56) and 1.10 (95% CI 0.97–1.26), respectively [15]. Several other studies found an elevated risk of hospital admission for acute MI in the first 7 days after laboratory-confirmed influenza. Combining the results, the incidence ratio ranges from 6.05 to 8.89 and is highest for older adults [16, 17]. Kwong et al. calculated that the incidence ratio for days 1–3 and days 4–7 amounts to 6.30 and 5.78, respectively. After 7 days, there is no significant increase in incidence. Also, compared with other respiratory viral diseases such as respiratory syncytial virus infection, the incidence ratio is highest for influenza A and B viruses (5.17 and 10.11, respectively) [17].

Several hypotheses exist that explain the ways influenza can trigger cardiovascular events, mainly through the activation of inflammatory and coagulation pathways [18–20]. This theory has been substantiated by a recent study, which showed that patients with elevated white blood cell and platelet counts are at higher risk of developing acute MI [16]. Influenza virus infection could lead to destabilisation of already susceptible atherosclerotic plaques, which could eventually result in coronary artery occlusion and thus acute MI. Another mechanism describes features of infection, which cause inadequate coronary perfusion through increased metabolic demand with fever, tachycardia and hypoxia [20, 21].

Once patients with diagnosed acute MI and concomitant influenza are admitted to hospital, they have worse outcomes than those with acute MI only. Possible causes are in-hospital death, development of shock, acute respiratory failure and acute kidney injury. Also, length of stay is increased in patients with acute MI and influenza compared with acute MI alone [22].

#### Effect of influenza vaccination on heart disease

In general, the effectiveness of the influenza vaccine depends on how well that vaccine matches the circulating influenza viruses strains in that particular season. In 2019–2020, vaccination effectiveness was 39% against any influenza illness, 45% against influenza B/Victoria illness and 30% against influenza A (H1N1)pdm09-associated illness in the United States [23]. Influenza vaccination resulted in a 26% reduction of the risk of ICU admission and a 31% reduction in the rate of death in case of influenza-associated hospitalisation [24].

Barnes et al. showed that the effectiveness of the influenza vaccine to protect against acute MI is about 29% (95% CI 9–44) [21]. This means the effectiveness is comparable to or even better than that of existing secondary preventive therapies, such as statins (prevention rate approximately 36%), antihypertensives (prevention rate approximately 15–18%), and smoking cessation (prevention rate approximately 26%). A meta-analysis showed that influenza vaccination results in a 36% reduction of major adverse cardiovascular events in patients at high risk of cardiovascular disease [25]. This preventive effect seems to be even more apparent in patients with recent acute coronary syndrome. Caldeira et al. identified a protective effect in the first 28 days after the influenza vaccine, which resulted in a decreased MI risk (relative risk 0.84, 95% CI 0.78–0.91) [26].

## Conclusion

Given the importance of influenza vaccination to prevent cardiovascular events, the cardiology community should be urged to actively promote vaccination. It is essential to remember that the beneficial effect of influenza vaccination is of the same magnitude as that of prescription of statins, antihypertensives, or cessation of smoking. Vaccination apathy in the cardiology community is therefore unacceptable.

Earlier this year, the American College of Cardiology published a health policy statement recommending COVID-19 vaccine prioritisation for heart disease patients with the highest risk [27]. This Point of View paper serves as a call to action to implement the flu vaccine in routine cardiac patient care. When promoting vaccination against influenza, barriers such as concerns about safety of the vaccine and low perceived risk of the disease should be discussed with patients to overcome vaccine hesitancy [28]. In addition, recent research has found that the best way to debunk misinformation is to first state the truth, then mention the false claim, followed by repeating the truth [29].

We hope that this viewpoint helps to urge cardiologists to actively advice their patients to get the seasonal influenza vaccination.
